# Expression of endogenous granzyme B in a subset of human primary breast carcinomas

**DOI:** 10.1038/sj.bjc.6601051

**Published:** 2003-07-01

**Authors:** S X Hu, S Wang, J P Wang, G B Mills, Y Zhou, H-J Xu

**Affiliations:** 1 Department of Molecular Therapeutics, The University of Texas MD Anderson Cancer Center, Houston, TX 77030, USA; 2 Department of Blood and Marrow Transplantation, The University of Texas MD Anderson Cancer Center, Houston, TX 77030, USA

**Keywords:** endogenous granzyme B, breast cancer, retinoblastoma tumour suppressor gene, nonimmune cells

## Abstract

Granzyme B (GrB) is the prototypic member of a serine protease family primarily used by cytotoxic lymphocytes to kill target cells. We report here that, by immunohistochemical staining of paraffin-embedded tumour sections, GrB protein was unexpectedly detected in malignant cells of a subset of breast cancers and their adjacent reactive endothelial and mesenchymal cells in which endogenous retinoblastoma protein (pRB) is overexpressed. The identity of the endogenous GrB was further confirmed experimentally in *RB*-deficient breast carcinoma cell culture upon overexpression of ectopic pRB. Our finding extends the recent paradigm-shifting trend for a more diverse biological role of granzyme B, and might provide a rational basis for exploring its potential prognostic value in a variety of human cancers.

Granzyme B (GrB) is a major component of cytoplasmic granules of cytotoxic T lymphocytes (CTL) and natural killer (NK) cells (Schmid and Weissmann, 1987; Trapani *et al*, 1988). In cellular immune reactions, GrB is produced by activated cytotoxic lymphocytes and initially stored in cytoplasmic granules. These granules are exocytosed, releasing GrB and other cytolytic proteins, including a pore-forming protein (perforin). GrB can enter target cells by autonomously crossing the cell membrane, or via a receptor-mediated pathway, and the coexistence of perforin facilitates intracellular trafficking of GrB in the target cells (Shi *et al*, 1997; Motyka *et al*, 2000). The critical role of cytotoxic lymphocyte GrB in target cell apoptosis has been well established (Heusel *et al*, 1994; Pinkoski *et al*, 2001). More recently, albeit controversially (Graubert *et al*, 1997), *GrB* expression has also been detected in other normal and malignant haematopoietic (nonlymphoid) cells. These include, for example, pluripotent stem cells capable of giving rise to all haematopoietic lineages (Hampson *et al*, 1992), CD34^+^ haematopoietic progenitor cells mobilised by chemotherapy and granulocyte colony-stimulating factor (Berthou *et al*, 1995), acute myeloblastic leukaemic cells under genotoxic stress (Bruno *et al*, 2000) and Kupffer cells (specialised macrophages in liver) (Tordjmann *et al*, 1998). In addition, activated keratinocytes are able to protect against invading pathogens through expression of endogenous GrB and perforin (Berthou *et al*, 1997), whereas perforin-independent expression of GrB and its specific inhibitor, proteinase inhibitor 9 (PI-9), in human testis and placenta may play a role in reproduction (Hirst *et al*, 2001). Also of relevance is a recent report showing that GrB was distributed in primary breast and lung cancer cells rather than in tumour-infiltrating lymphocytes (TILs) (Kontani *et al*, 2001). In the latter study, the authors assume cancer cells may acquire GrB released from TIL. These observations inspire speculation that under certain circumstance, endogenous GrB may be expressed directly in nonhaematopoietic tumour cells, which is strongly supported by the results of our present work. We also report for the first time that there is apparent correlation between endogenous GrB and pRB expression in breast tumour specimens.

## MATERIALS AND METHODS

### Tumour specimens

Paraffin-embedded tissue sections of primary breast carcinoma specimens were obtained from the archives of the Department of Pathology at The University of Texas MD Anderson Cancer Center. They were randomly selected among cases there were plenty of tissues available from the tumour bank, and patients who had been operated before other therapy.

### Immunochemistry and Western blotting

Immunohistochemical staining of pRB and GrB proteins in breast tumours was carried out on formalin-fixed, paraffin-embedded tissue sections with an antigen retrieval protocol as previously described (Xu, 1995; Yakirevich *et al*, 1999). The anti-RB antibody RB-WL-1 (Xu, 1995) and anti-GrB monoclonal antibody B18.1 or GrB7 (Alexis) were used. After antibody binding, the slides were processed for colour development using the avidin–biotinylated peroxidase method, and were counterstained with Mayer's haemotoxylin. All slides were coded, and the pRB and GrB staining results were scored separately by two investigators. Immunostaining and Western blotting detection of pRB and GrB proteins in cultured cells were done as described (Zhou *et al*, 1994; Berthou *et al*, 1997; Pinkoski *et al*, 2000). For double immunofluorescence analysis of pRB and endogenous GrB, anti-pRB monoclonal antibodies G3-245 (FITC-labelled mouse IgG1; PharMingen, SanDiego, CA, USA) and B18.1 (Texas Red-labelled mouse IgG2a) were used. Digital images were acquired by confocal laser scanning microscopy (CLSM) (Zeiss LSM 210, Thornwood, NY, USA).

### Ribonuclease protection assay (RPA) and Northern blotting

A multiprobe template set for human apoptosis-related genes (hAPO-1; PharMingen) was used for RPA, which was done according to the manuals. Northern blot analysis was conducted on total RNA (PBL) or mRNA (MDA-MB-468) samples (2 *μ*g lane^−1^), using ^32^P-labelled full-length *GrB* cDNA probe and *β*-actin cDNA probe (for rehybridisation). After exposure to a storage phosphor screen, quantitative comparison of endogenous *GrB* transcript levels between the samples was carried out by using a Storm 860 PhosphorImager system and ImageQuant software (Molecular Dynamics, Piscataway, NJ, USA), and fold increases in transcription were calculated based on the PhosphorImager counts of *GrB* bands adjusted for counts of *β*-actin bands from the same samples.

### Deglycosylation of GrB proteins

The conditions for hydrolysis of *N*-glycosylated proteins in whole cell lysates by Endo H (40 U mg^−1^, Boehringer, Mannheim, Germany) were optimised based on the general guidelines previously established for deglycosylation of purified glycoproteins (Trimble and Maley, 1984). Briefly, cell extracts were prepared in 50 mM Tris·Cl (pH 8.0) containing 120 mM NaCl and 0.5% NP-40, and then changed into 100 mM phosphate reaction buffer (pH 5.8) by using Bio-Spin 6 columns (Bio-Rad, Hercules, CA, USA). In the case of using denatured substrates, the cell extracts were preheated for 2 min at 100°C with 1.2-fold weight excess of sodium dodecyl sulphate (SDS) in relation to the protein contents. The deglycosylation assays were carried out in triplicate in microfuge tubes. Each tube contained 5 *μ*g of total cellular proteins in 25 *μ*l reaction buffer, a cocktail of proteinase inhibitors, 100 mM of *β*-mercaptoethanol (*β*-ME), and without Endo H (Tube 1, Control), or with Endo H (Tubes 2 and 3). Cell extracts in Tube 3 were predenatured. All reaction mixtures were incubated for 18 h at 37°C, and then analysed by Western blotting as described above. For *in vivo* deglycosylation, cells were cultured in the presence of tunicamycin (5 *μ*g ml^−1^, Sigma, St Louis, MO, USA) overnight.

## RESULTS

### Detection of endogenous GrB in primary breast carcinomas overexpressing pRB

In phytohaemagglutinin (PHA)-stimulated peripheral blood lymphocytes (PBL), coincident with the strong induction of *GrB* expression (Schmid and Weissmann, 1987; Trapani *et al*, 1988), pRB protein increased eight-fold, and *RB* RNA levels increased 2–4-fold (Furukawa *et al*, 1990). The same appears to hold for the primary keratinocytes in culture. While human keratinocytes grown *in vitro* reportedly synthesised endogenous GrB protein (Berthou *et al*, 1997), we observed that these activated keratinocytes also had elevated pRB expression (data not shown). Therefore, to address the issue of whether breast cancer cells can produce their own GrB, a total of 25 randomly selected breast carcinomas were examined for endogenous pRB and GrB expression.

**Figure 1:**
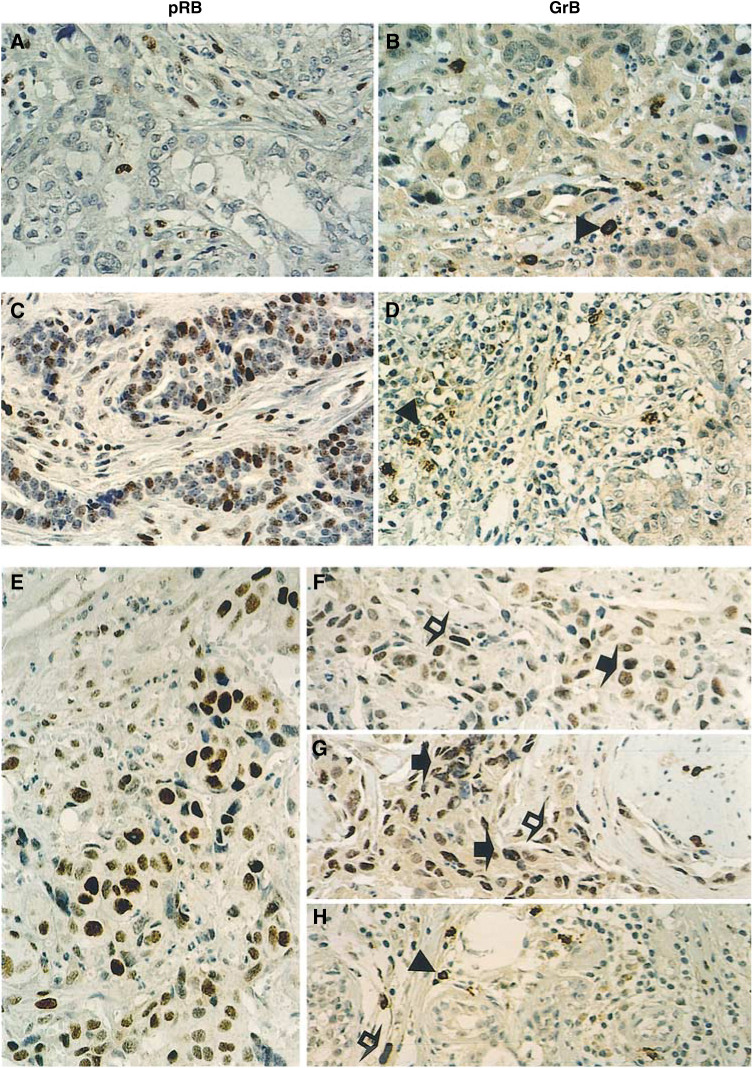
Detection of endogenous GrB in primary breast carcinomas overexpressing pRB (pRB^++^) by immunohistochemical staining of paraffin-embedded tissue sections. (**A**, **C**, **E**) pRB staining, showing typical pRB^−^ (**A**), pRB^+^ (**C**), and pRB^++^ (**E**) tumours. Note that the tumour cells in panel **E** (pRB^++^) display uniformly strong pRB staining, while the tumour cells in panel (**C**) (pRB^+^) show nuclear staining heterogeneity of the RB protein, ranging from quite positive to seemingly negative (Xu, 1995). (**B**, **D**, and **F**–**H**) The same tumours corresponding to the left panels were stained for GrB. Panels **B** and **D**, in either pRB^−^ (**B**) or pRB^+^ (**D**) tumours, malignant cells are GrB negative, but some infiltrating lymphocytes are GrB^+^. Panels (**F**–**H**), representative areas of the same pRB^++^ tumour shown in Panels **E**. GrB^+^ tumour cells (**F**, **G**), or lymphocytes (**H**) were evident. Note the finely granular distribution of endogenous GrB protein in tumour cells of panel (**G**). Arrowheads, GrB^+^ lymphocytes; solid arrows, GrB^+^ tumour cells; open arrows, GrB^;^ mesenchymal and endothelial cells. Scale bar, 50 *μ*m.

As illustrated in Figure 1A

, by immunohistochemical staining of routinely processed pathological specimens, we found that five of the 25 breast tumours were pRB^−^, that is, loss of pRB staining occurred in every malignant cell of the tumours. In these pRB^−^ tumours, some (but not all) of the reactive stromal cells were stained positively for pRB, which was consistent with the view ;that expression of pRB in normal tissues was regulated by their proliferation and differentiation states (Xu *et al*, 1991a, 1991b; Shan *et al*, 1994; Cordon-Cardo and Richon, 1994). Malignant cells of the five pRB^−^ breast tumours were all negative for GrB, although there were clearly GrB^+^ TILs in immediately adjacent tumour stroma; the latter served as an excellent internal control for validating the GrB staining (Figure 1B). Second, 17 of the 25 cases fulfilled the established criteria for pRB^+^ tumours (Cance *et al*, 1990; Xu *et al*, 1991a, 1991b; Xu, 1995), that is, the observed pRB immunoreactivity patterns in these tumours were highly heterogenous, and the staining intensity was not uniform among the tumour cells, with more or less of the tumour cell nuclei stained positively (Figure 1C). In the majority (16 out of 17) of the pRB^+^ cases, all tumour cells stained negatively for GrB, while TILs in the same tumour sections were GrB^+^ (Figure 1D). Third, the remaining three tumours expressed extremely high levels of pRB as determined by their uniformly high intensity of pRB staining (pRB^++^, Figure 1E) (Cote *et al*, 1998). In these three pRB^++^ and one pRB^+^ tumours, GrB staining was readily detected in many tumour cells as well as in nonlymphoid reactive stromal cells, including endothelial and mesenchymal cells (Figure 1F, G). The intensity of the endogenous GrB staining was variable, with some areas exhibiting typical granular or dot-like cytoplasmic and nuclear staining (Figure 1G). In this small cohort study, the correlation between endogenous GrB and pRB protein expression in malignant cells appeared to be very significant (*P*<0.001), although an inadequate sample size for statistical calculation has precluded a more definitive conclusion (Table 1

**Table 1:** Correlation between endogenous GrB and pRB protein expression in breast cancers

	**Endogenous GrB expression**	
**pRB status**	**GrB^−^**	**GrB^+^**
pRB^−^ (*n*=5)	5	0
pRB^+^ (*n*=17)	16	1
pRB^++^ (*n*=3)	0	3

Values are number of specimens. The correlation between GrB and pRB expression was significant (*P*<0.001, calculated using the *χ*^2^ method). Computation was performed with the STATA statistical software (Computing Resource Center, Santa Monica, CA, USA).

). On the other hand, the number of TILs with GrB^+^ staining was variable within the same cohort, and in general was unrelated to the pRB status of the tumour specimens (Figure 1B, D and H). Two proven anti-granzyme B antibodies, B18.1 (Berthou *et al*, 1997) and GrB7 (Kummer *et al*, 1993), were used for the studies with essentially similar results.

### Evidence from culture: confirmation of endogenous *GrB* expression in breast cancer cells

Until recently, expression of *GrB* has been implicated solely in lymphoid cells. The discovery of GrB in nonlymphoid cells, such as mobilised haematopoietic CD34^+^ progenitor cells, epidermal keratinocytes, testis and placenta (Berthou *et al*, 1995, 1997; Hirst *et al*, 2001), challenged the existing, perhaps oversimplified model for GrB function. In these latter studies, however, expression of the *GrB* mRNA was demonstrated only by *in situ* hybridisation using antisense *GrB* RNA probes. The method was not able to determine the extent of the nucleotide sequence identity between more or less closely related RNA species, nor the size of the transcripts. An independent study reported by others failed to detect *GrB* mRNA expression in mobilised haematopoietic CD34^+^ progenitor cells when an S1 nuclease protection assay was employed (Graubert *et al*, 1997).

To further validate our finding of endogenous GrB in primary breast cancer, we conducted *in vitro* experiments to confirm the identity of the GrB protein and messenger RNA, and the correlation between induction of endogenous GrB and overexpression of pRB. This was accomplished by using a panel of *RB*-reconstituted MDA-MB-468 breast carcinoma cell lines (Xu *et al*, 1997) in which expression of pRB is tightly controlled by tetracycline (Tc). The ectopic RB protein expressed in these cell clones reach the highest level about 24 h after removal of Tc from the cell culture medium, and will then become totally dephosphorylated within 24–40 h (Xu *et al*, 1997). The levels of pRB expression in these cultured cells in Tc-free medium were comparable to the elevated pRB expression in subsets of primary tumours (Xu, 1995; Xu *et al*, 1997; Cote *et al*, 1998 and compare Figure 1E with 3A

**Figure 3:**
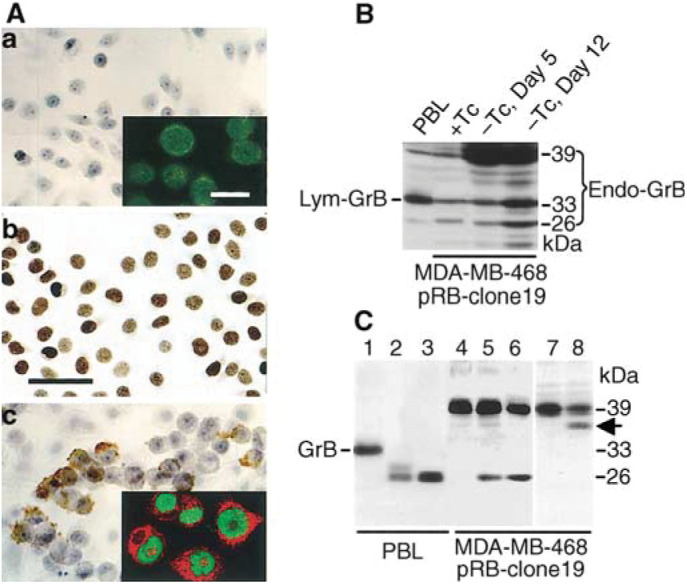
Characterization of endogenous GrB protein in *RB*-reconstituted MDA-MB-468 tumour cells. (**A**) Immunochemical staining of Endo-GrB (panels *a* and *c*) and pRB (panel b) of MDA-MB-468 pRB-clone 19-4. Endo-GrB was not detectable in tumour cells cultured in Tc-containing medium (Panel a), but was induced in Tc-free medium (Panel c). Tumour cells in Tc-free medium for 2 days exhibited uniformly pRB^+^ staining (Panel b). The CLSM images shown in the inserts of Panels a and c illustrate the double immunofluorescence staining of pRB (FITC, green) and Endo-GrB (Texas Red). Scale bars, 25 *μ*m (12.5 *μ*m in insets). (**B**) Western blotting. Endo-GrB protein triplets with molecular weights of 26, 33, 39 kDa were accumulated in *RB*-reconstituted cells grown in Tc-free medium. (**C**) The deglycosylated Endo-GrB and Lym-GrB proteins are identical in apparent molecular masses. Cell lysates were prepared from IL-2-activated PBL or MDA-MB-468 pRB-clone 19 cells (in Tc-free medium, Day 5). Each lane contains 5 *μ*g of total cellular proteins treated with (lanes 1 and 4) reaction buffer only, and (lanes 2, 3, 5 and 6) with Endo H. Cell extracts in lanes 3 and 6 were predenatured. Following deglycosylation, both the 33-kDa mature Lym-GrB protein (lane 1) and the 39-kDa Endo-GrB protein (lanes 4) migrated to the identical position with an apparent *M*_r_ of 26 kDa (lanes 2, 3, 5 and 6). Also note that when small amounts of total cellular proteins (5 *μ*g) were loaded in each lane, only the major species, that is, the 33-kDa glycosylated Lym-GrB in lane 1 and the 39-kDa glycosylated Endo-GrB in lane 4 were visible prior to Endo H treatment. (Lanes 7 and 8) The *RB*-reconstituted MDA-MB-468 cells were cultured in the absence (lane 7) or presence (lane 8) of tunicamycin. Arrow indicates a partially deglycosylated Endo-GrB of ∼36 kDa.

, panel b below). Both ribonuclease protection assay (RPA) and Northern blot analysis showed that the endogenous *GrB* mRNA levels were increased up to 16-fold in *RB*-reconstituted MDA-MB-468 tumour cells after removal of Tc for 4 days (Figure 2

**Figure 2:**
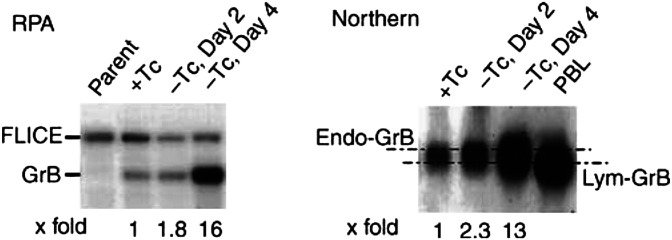
Transcriptional upregulation of endogenous *GrB* in *RB*-reconstituted MDA-MB-468 breast carcinoma cells. RPA and Northern blotting analyses were performed on RNAs extracted from parental MDA-MB-468 (pRB^−^) and a representative Tc-regulated *RB*-reconstituted clone at each indicated day. (+Tc) medium containing 0.5 *μ*g ml^−1^ of Tc; (−Tc) Tc-free medium. An RNA sample from PBL of healthy donors cultured in the presence of 50 U ml^−1^ of IL-2 was included in the Northern blot as *GrB*-positive control. Note that *Endo-GrB* mRNAs are slightly larger than the *Lym-GrB* mRNA on the Northern blot. The numbers under the blots indicate the fold increases in *Endo-GrB* transcription. Similar results were obtained with three independent clones of *RB*-reconstituted MDA-MB-468 breast carcinoma cells (data not shown).

). The Northern blotting results also suggested that the endogenous *GrB* mRNA detected in breast tumour cells was slightly larger than the *GrB* mRNA from IL-2-stimulated peripheral blood lymphocytes (PBL) (Figure 2). The difference in transcript sizes is owing to differential usage of upstream transcription start sites at the *GrB* locus (Xu HJ *et al*, unpublished data).

Immunocytochemistry and double immunofluorescence staining of pRB and GrB showed that endogenous GrB protein (Endo-GrB) was accumulated in the pRB^+^ (in Tc-free medium), but not in the pRB^−^ (in medium containing 0.5 *μ*g ml^−1^ of Tc) MDA-MB-468 tumour cells (Figure 3A). Endo-GrB was located both in cytoplasm and nuclei of the *RB*-reconstituted tumour cells (Figure 3A); a comparable pattern was observed for IL-2-activated PBL GrB (Lym-GrB). Western blotting revealed Endo-GrB protein triplets from the *RB*-reconstituted MDA-MB-468 cells with apparent molecular masses (*M*_r_) of 26, 33 and 39 kDa (Figure 3B). By SDS–PAGE, the 33-kDa Endo-GrB protein band is identical to the mature glycosylated Lym-GrB protein from human PBL. The 39-kDa protein was markedly increased in the tumour cells after overexpression of pRB. An *in vitro* hydrolysis assay using endoglycosidase H (Endo H) revealed that, after deglycosylation, the 39-kDa Endo-GrB and the 33-kDa Lym-GrB migrated to an identical position corresponding to a reduced *M*_r_ of 26 kDa (Figure 3C). When the *RB*-reconstituted MDA-MB-468 cells were cultured in the presence of tunicamycin, an inhibitor of glycosylation, a partially deglycosylated 39-kDa Endo-GrB protein appeared at ∼36 kDa (Figure 3C, lane 8). Human *GrB* cDNA contains a single open reading frame encoding a preproenzyme of 247 amino acids. The predicted mature Lym-GrB is an active enzyme of 227 amino acids (after N-terminal cleavage by signal peptidase and dipeptidyl peptidase I [DPPI]) with an unglycosylated *M*_r_ of 26 kDa. Marked differences in apparent *M*_r_ of the mature Lym-GrB, however, have been reported in the literature, between 26 and 67 kDa, which are usually interpreted as being due to heterogeneous N-linked glycosylation (Trapani *et al*, 1994). Both glycosylated and nonglycosylated mature Lym-GrB are proteolytically active.

## DISCUSSION

In summary, we observed Endo-GrB expression in primary breast cancer cells, which appeared to be coincident with overexpression of pRB. Expression of endogenous GrB can also be manipulated experimentally by making *RB*-deficient breast tumour cells overexpressing ectopic pRB in culture. The identity of the endo-GrB was confirmed by immunochemistry, Northern blotting, Western blotting and deglycosylation. The mechanism of endo-GrB expression in breast cancer cells have yet to be established. We noticed that in paraffin-embedded breast tumour sections, germinal centres of reactive lymph nodes showed intense pRB staining, but were GrB negative (data not shown). Therefore, it seems unlikely that pRB directly regulates *GrB* promoter in breast cancers; it is more likely that the overexpression of pRB mediates senescent arrest and/or terminal differentiation (Hinds and Weinberg, 1994; Xu *et al*, 1997), resulting in endo-GrB expression that occurs prior to the onset of the postsenescent apoptosis of the tumour cells. In this regard, Kontani *et al* have reported that the percentage of breast and lung cancer cells with positive GrB immunoreactivity (as mentioned above, the authors had assumed cancer cells acquired GrB released from TIL) was inversely correlated with regional lymph node metastasis (Kontani *et al*, 2001). While overexpression of pRB in primary tumours is not invariably associated with better prognosis (Cote *et al*, 1998), the finding of an intrinsic GrB directly expressed by human nonlymphoid tumour cells adds a new dimension to the clinical research on potential prognostic value of pRB expression in primary cancers. Furthermore, the diverse biological presence of GrB might be complementary to the existing paradigm for cytotoxic lymphocyte-mediated target cell death, allowing synergistic interactions between the local mechanism of defense and the immune system. We can thus look forward to intense research on both the theoretical and practical implications of endogenous GrB expression in nonlymphoid cells, which may lead us in an unexpected direction.
